# Ulcerating skin lesions from blastic plasmacytoid dendritic cell neoplasm responding to low-dose radiotherapy—a case report and literature review

**DOI:** 10.1007/s00066-024-02200-2

**Published:** 2024-01-29

**Authors:** Elgin Hoffmann, Simon Böke, Chiara De-Colle, Claudia Lengerke, Karim-Maximilian Niyazi, Cihan Gani

**Affiliations:** 1 University Hospital for Radiotherapy, University Hospital Tübingen, 72076 Tübingen, Germany; 2 Department of Internal Medicine II, Hematology, Oncology, Clinical Immunology and Rheumatology, University Hospital Tübingen, 72076 Tübingen, Germany

**Keywords:** Blastic plasmacytoid dendritic cell neoplasm, Palliative radiotherapy, Hematological malignancy, Low-dose radiotherapy, Skin lesions in hematological malignancies

## Abstract

Blastic plasmacytoid dendritic cell neoplasm (BPDCN) is a rare hematologic malignancy that can manifest with skin nodules and erythematous plaques. In most cases BPDCN progresses rapidly, causing multiple skin lesions and also affecting internal organs and bone marrow, warranting initiation of systemic therapies or hematopoietic stem cell transplantation (HCT). Although not curative, radiotherapy for isolated lesions might be indicated in case of (imminent) ulceration and large or symptomatic lesions. To this end, doses of 27.0–51.0 Gy have been reported. Here, we present the case of an 80-year-old male with BPDCN with multiple large, nodular, and ulcerating lesions of the thorax, abdomen, and face. Low-dose radiotherapy of 2 × 4.0 Gy was administered to several lesions, which resolved completely within 1 week with only light residual hyperpigmentation of the skin in affected areas and reliably prevented further ulceration. Radiotoxicity was not reported. Therefore, low-dose radiotherapy can be an effective and low-key treatment in selected cases of BPDCN, especially in a palliative setting, with a favorable toxicity profile.

## Introduction

Blastic plasmacytoid dendritic cell neoplasm (BPDCN) is a rare hematologic malignancy arising from precursor plasmacytoid dendritic cells [1, 2]. Multiple nodular and sometimes ulcerating skin lesions which can encompass the whole integument [3] are often the first manifestation. Internal organs, the lymphatic system, and bone marrow can also be affected [2, 4–6]. In a palliative setting, extensive skin lesions can cause significant symptomatic strain for patients and favorable results of local radiotherapy have been reported. So far, the literature on this disease entity reports higher radiation doses to the skin of up to 51.0 Gy in 3.0 Gy per fraction, corresponding to several weeks of treatment time. Here, we report a patient with a complete and lasting response to a short and hypofractionated electron radiotherapy dose of 8.0 Gy in 4.0 Gy per fraction, resulting in a significantly shorter treatment time in a palliative setting.

## Case report

**Fig. 1:**
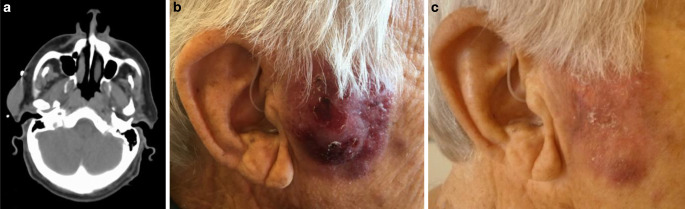
**a** Planning CT scan showing the preauricular nodule on the right side. As the localization was superficial, electron therapy was chosen. The CT scan was used to measure the thickness of the irradiated lesion, according to which the electron energy was chosen. **b** Clinical presentation of the preauricular nodule. No pain was reported, but secretion had occurred earlier. Localization in the face and thus visibility of the nodule was stressful for the patient. **c** Clinical response 1 week after irradiation. Slight discoloration of the skin can still be observed. Secretion has stopped and the prominence of the lesion has completely resolved

**Fig. 2:**
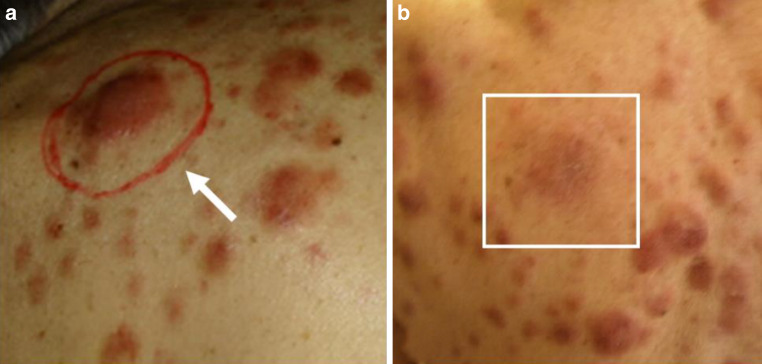
**a** Image of a second lesion, intermittently exhibiting secretion, below the right scapula preradiotherapy. The lesion has been marked to facilitate set-up for electron beam irradiation (*white arrow*). Radiotherapy in this case was conducted using a round tube frame. A 1-cm margin was chosen between the lesion and the tube. **b** Clinical outcome 4 weeks after radiotherapy (irradiated lesion marked by *white square*). No secretion reoccurred after irradiation and only discoloration of the skin can be observed

An 80-year-old male with BPDCN with nodular, indolent cutaneous lesions of the thorax, abdomen, and face was referred to our department. He reported a history of chronic myelomonocytic leukemia (CMML) which had been diagnosed 18 years previously and for which a watch-and-wait strategy had been adopted. Associated BPDCN was histologically confirmed as indolent skin lesions surfaced on the back and right temple (Figure 1 and 2). Dermal histopathology described diffuse infiltration of blast cells with increased mitotic activity (Ki67 60–70%). Atypical lymphoid cells showed positive expression of Bcl-2, CD56, CD123, and, to a lesser extent, CD4. No expression of CD3, CD20, CD30, or MPO was found, confirming the diagnosis of BPDCN skin involvement. Bone marrow analysis at the time of diagnosis showed accelerated CMML with an increased blastic fraction (7.5%), but no infiltration of BPDCN. A CT scan yielded splenomegaly and lymphadenopathy without involvement of internal organs. As BPDCN has been reported secondary to CMML, systemic therapy with six cycles of azacitidine, an analogue of cytidine, was initiated. Under azacitidine treatment, the CMML responded well with normalization of thrombocyte and monocytic cell count, but BPDCN lesions progressed. Therefore, targeted therapy with tagraxofusp, a fusion antibody binding interleukin 3 (IL-3), was initiated. However, treatment was discontinued after two cycles as BPDCN lesions showed further progression and treatment with hydroxyurea was initiated. At this point, multiple skin lesions developed, affecting mainly the face, thorax, and abdomen, and the patient was referred to our department. Laboratory findings showed a leukocyte count of 4480/µl, with an increased monocyte count (36.4%) of atypical immature monocytes and a reduction in neutrophils (18.5%). Thrombocyte counts were within the normal range (161,000/µl). Hemoglobin was 11.7 g/dl and both rouleaux and acanthocytes were found. Radiotherapy was considered because of ulceration and secretion of both a preauricular skin nodule measuring 50 × 40 mm and two rapidly progressing dorsal thoracic lesions bothering the patient. All three superficial lesions were treated with electron beams (the facial nodule with 8 MeV and the others with 6 MeV), delivered in 2 × 4.0 Gy and a 10-mm bolus on subsequent days (for a detailed description, please refer to Table 1). Within 7 days, ulcerations had healed and skin protuberance of the lesions had resolved, showing only slight erythematous maculae of the skin (Figure 1 and 2). Secretion had ceased completely. No radiation-associated toxicities were reported. The patient expressed his satisfaction with the outcome yielded by radiotherapy. Therefore, four further thoracic lesions were also treated with 8 MeV in 2 × 4.0 Gy to prevent ulceration following initial radiotherapy (Table 1). Here, an equally good response at 1 and 4 weeks after radiotherapy could be observed. Under treatment with hydroxyurea, non-irradiated lesions did not respond. Ultimately, venetoclax was started due to systemic progression of BPDCN with bone marrow affectation. However, bone marrow affectation progressed after 1 month despite venetoclax treatment, with the patient developing pancytopenia, gastric hemorrhage, and cerebral symptoms in the form of progressive confusion. According to the patient’s wishes, best supportive care was initiated following his discharge from hospital. Up to discharge from our hospital, irradiated lesions showed a durable response.

**Table 1:** First treatment was initiated after skin lesion progression under treatment with hydroxyurea due to the symptoms detailed in the text. After completion of radiotherapy, systemic treatment was switched to venetoclax due to systemic progression, with no effect on non-irradiated lesions. For all lesions, a 1-cm bolus and a 1-cm margin around the lesion to the frame/tube was chosen to allow for dose build-up. Electron energy was chosen depending on the prominence of the lesion and was calculated using percentage depth–dose curves aiming at a dose maximum at the surface of the skin and coverage of the lesion with at least 80% of the applied dose

Site	Symptoms	Fractionation	Electron energy	Set-up	Outcome	Toxicity
*First treatment*						
Right cheek	Secretion, large nodule, cosmetic concerns	2 × 4 Gy	8 MeV	Square 6 × 6 cm frame, 1 cm bolus	Cessation of secretion, regression of the nodule, slight residual discoloration of the skin	None reported
Right shoulder (below scapula)	Secretion, large nodule bothering patient	2 × 4 Gy	6 MeV	Round tube, 5 cm diameter, 1 cm bolus	Cessation of secretion, regression of the nodule, slight residual discoloration of the skin	None reported
Lower back	Large nodule, hindering lying in a supine position	2 × 4 Gy	6 MeV	Round tube, 5 cm diameter, 1 cm bolus	Regression of the nodule residual discoloration of the skin	None reported
*Second treatment*						
Epigastric area	Three large nodules with secretion	2 × 4 Gy	8 MeV	Square 10 × 10 cm frame, 1 cm bolus	Cessation of secretion, regression of the nodules, slight residual discoloration of the skin	None reported
Right thorax, sub mammary	Large nodule bothering patient	2 × 4 Gy	8 MeV	Square 6 × 6 cm frame, 1 cm bolus	Cessation of secretion, regression of the nodule, slight residual discoloration of the skin	None reported

## Discussion

Blastic plasmacytoid dendritic cell neoplasm (BPDCN) is a rare and aggressive hematological neoplasm characterized by CD123+ (IL‑3 receptor) expression and at least one plasmacytoid dendritic cell marker in addition to expression of CD4+ and CD56+. Other markers that consistently show positive staining include CD43, CD45, and Bcl-2 (as in our case), CD2AP, and markers associated with plasmacytoid dendritic cell origin like HLA-DR, CD303+, CD304+, and cTCL1+. Negative staining is found for CD3, CD14, CD19, PAX5, lysozyme, myeloperoxidase, and CD34 [15] according to the current WHO classification 2022 [1]. Typically, a high Ki-67 proliferation index is found. In case of skin involvement, infiltration of the dermis and subcutis by immature blastoid neoplastic cells is observed in dermal histology. In 10–20% of cases, it is associated with other hematologic neoplasms, and can arise from myeloid neoplasms like CMML and acute myeloid leukemia (AML) [16–18]. Systemic therapy in patients with good performance status encompasses regimens analogous to induction therapy in acute leukemia (such as ALL/LBL or AML protocols) and a moderate intense non-Hodgkin lymphoma regimen (like cyclophosphamide, doxorubicin, vincristine, and prednisone [CHOP]) [8, 10, 19]. A meta-analysis published by Bruch et al. provided evidence for the combination of allogenic stem cell transplantation after myeloablative conditioning with total body irradiation in a curative setting [9]. reporting improvement in both overall and progression-free survival. However, as BPDCN with a median age at onset of 65 years mostly affects elderly patients, many are not eligible for the intense polychemotherapy regimens needed for curative treatment, so less intense regimens or monotherapies are chosen. Due to the rarity of BPDCN, no specific recommendation can be made, but there is evidence for a number of substances including etoposide, hydroxyurea, and azacitidine [5, 20]. Newer and targeted treatment options include tagraxofusp [21–24], which selectively binds the IL‑3 receptor which is expressed abundantly in BPDCN [25], and venetoclax, an oral Bcl‑2 inhibitor, approved for chronic lymphocytic leukemia [6, 7, 26, 27]. Although initial response to systemic treatment is usually good, BPDCN shows a tendency for early relapses, associated with a dismal prognosis with a 2-year survival rate below 20% and thus the need for palliative treatment options.

Literature on radiotherapy in BPDCN is scarce, with only few reports detailing radiotherapy regimens, regardless of curative or palliative settings. In our literature review, we identified 19 publications with which reported on local radiotherapy as the only or part of the first-line treatment in 47 patients with cutaneous BPDCN lesions (Table 2; [11, 13, 14, 28–43]). With the exception of five patients aged 23, 32, 36, 38 and 43 years, the age range was 59–90 years, corresponding to the median age of onset of 65 years. As this older patient cohort is often not eligible for high-dose chemotherapy, in most cases radiotherapy was chosen as the only first-line treatment or as a complement to intensity-reduced chemotherapy regimens. The gender ratio was 20 male to 10 female patients; however, gender was not reported in all publications. Likewise, most publications did not detail dose, set-up, or target volume for local radiotherapy. The most detailed publication dedicated to radiotherapy dose and delivery in BPDCN is by Ishibashi et al. [11], who reported treatment of a patient with several skin nodules who had declined chemotherapy. In their case, the patient was treated with 30 Gy in 10 fractions using electron beam irradiation to an isolated painful lesion on the left forearm, with a good response of the irradiated but progression of the non-irradiated lesions. All other publications also describe favorable responses to radiotherapy, with an at least transient partial or complete response of irradiated cutaneous nodules. Of the few publications giving detail on radiation protocols, one reported a cumulative dose of 27.0 in single fractions of 3.0 Gy [14], to which a complete response could initially be observed. However, relapse occurred 2 months after radiation treatment. In a case report by Fontaine et al., the patient received 40 Gy in combination with methotrexate and L‑asparaginase, resulting in a complete response and the patient being alive 30 months after treatment completion [30], and Amitay-Laish describe 2 patients who showed a complete and lasting response to consolidative radiotherapy with 36 Gy [43]. In those cases, however, high-dose chemotherapy was applied as the patients were aged 38 and 23 years old. A similar good result was attained in the case of a 32-year-old male who also received consolidative radiotherapy with 36 Gy after CHOEP-14 [41]. In two other reports where comparable doses were chosen—34 Gy and two patients receiving 40 Gy—in combination with chemotherapy, however, only a partial response with rapid systemic progression and a relapse-free survival of less than 12 months, respectively, could be observed [40, 42]. It is noteworthy that lasting remissions could be attained in younger patients, probably corresponding to an eligibility for high-dose chemotherapy regimens and stem cell transplantation, but possibly also due to a different tumor biology in younger patients. Three publications describe higher cumulative doses of 50.0–51.0 Gy [32, 37, 39]. In all of these cases, an initial complete response could be attained. However, only in one case, reported by Higgins et al., did radiotherapy as the only treatment lead to long-term disease remission [39]. In the two other patients, death from systemic progression occurred at 9 months and 25 months, respectively, after radiotherapy [37, 39]. In cases in which radiotherapy doses were not detailed, mixed responses to radiotherapy were reported. In general, local radiotherapy led to a partial or complete remission of irradiated lesions, but most patients showed (systemic) progression, with a range of relapse-free survival of 2–31 months (Table 2). Even in an early stage of the disease where only dermal involvement is found, higher doses to single or singular lesions did not consistently lead to lasting remission or prevent systemic progression [32, 37]. It might be hypothesized that the combination of local radiotherapy with chemotherapy might lead to longer remission [35], especially in younger patients [43]. However, not all patients receiving additional chemotherapy showed longer remission [29, 31] and the presented data are not reliable enough to make a recommendation for combination therapy, especially as crucial details on radiation dose and toxicity profile are missing in most reports and heterogenous systemic therapies were applied. Nevertheless, as older and frail patients are often not suitable for high-dose chemotherapy, higher radiation doses in cases of single dermal lesions without systemic involvement might provide a suitable treatment option—either as single therapy or in combination with dose-reduced chemotherapy regimens—to provide local control with limited toxicity and prolong relapse-free survival in this patient clientele.

**Table 2:** Overview of publications reporting radiotherapy for blastic plasmacytoid dendritic cell neoplasm (BPDCN) as a (part of) first-line treatment

Author	Publication date	Number of patients	Patient characteristics	Clinical presentation	Systemic involvement	Treatment	Radiotherapy	Outcome
Bekkenk [28]	2004	8	Not specified	Not specified	Not specified	Not specified	Not specified	Not specified
Petrella [29]	2005	6	43–82 years old; 5 males/1 females	Different stages of disease	Different stages of disease	LRT; in three cases combination with polychemotherapy	Not specified	Relapse-free survival 2–31 months
Fontaine [30]	2009	1	64-year-old female	Singular cutaneous lesion	No	LRT + methotrexate/L-asparaginase	40 Gy	Complete remission, patient alive after 30 months
Kaune [40]	2009	1	66-year-old female	Single cutaneous lesion	No	Cyclophosphamide, hydroxydaunorubicin, vincristine, prednisone + LRT	34 Gy	Partial response, systemic progression 6 weeks after radiotherapy
Xue [13]	2010	1	36-year-old male	Several cutaneous lesion	No	RT + prednisolone	Not specified	Partial response, received stem cell transplant afterwards
Dalle [31]	2010	6	60–83 years old; 3 males/3 females	Single cutaneous lesions	No	LRT; in one case combined with methotrexate/L-asparaginase	Not specified	Relapse-free survival 2–11 months
Miyashita [32]	2011	1	77-year-old male	Single cutaneous lesion	No	LRT	51 Gy in 3 Gy fractions	Relapse-free survival 10 months, death after 25 months
Dohm [41]	2011	1	32-year-old male	Single cutaneous lesion	No	CHOEP-14	36 Gy	Complete remission, relapse-free survival 11 months
Pileri [33]	2012	5	Not specified	Single cutaneous lesions	No	LRT	Information not available	Median survival of 23 months
Hashikawa [34]	2012	4	59–90 years old; 2 males/2 females	Different stages of disease	Different stages of disease	LRT; in two cases with either prednisone or VP-16	Not specified	Relapse in two cases, one missing information, one durable response
Tsunoda [14]	2012	1	74-year-old male	Single cutaneous lesion	No	LRT	27 Gy in 3 Gy fractions	Initially complete response, relapse after 2 months
Sugimoto [35]	2013	1	74-year-old male	Single cutaneous lesion	No	LRT + dexamethasone, VP16, ifosfamide, carboplatin	Not specified	Complete response; no evidence of disease at 12 months
Pagano [36]	2013	2	Not specified	Multiple cutaneous lesions	Yes	LRT in addition to induction chemotherapy before stem cell transplant	Not specified	Response to radiotherapy not detailed
Yu [37]	2015	1	79-year-old male	Two isolated skin lesions	Not initially	LRT	50 Gy in 2 Gy fractions	Death of patient of multiorgan failure 9 months later
Heinicke [42]	2015	2	62-year-old female, 79-year-old male	Single cutaneous lesion	No	Patient 1: vincristine, prednisolone, followed by CHOP + LTR; patient 2: primary LTR	40 Gy	Patient 1: complete remission, relapse within 1 year; patient 2: complete response, relapse-free survival 6 months
Ishibashi [11]	2015	1	77-year-old male	Multiple cutaneous lesions	No	LRT; chemotherapy was declined	30 Gy in 3‑Gy fractions	Irradiated lesions responded well, progression of untreated lesions
Amitay-Laish [43]	2017	2	38-year-old male, 23-year-old female	Single cutaneous lesion	No	Hyper-CVAD, alternating with methotrexate and cytarabine + consolidative LRT	36 Gy	Relapse-free survival at 72 and 108 months, respectively
Brüggen [38]	2020	2	Not specified	Not specified	Yes	LRT	Not specified	1 patient with initial partial response, who later progressed; 1 patient with progressive disease
Higgins [39]	2022	1	84-year-old male	Single cutaneous lesion	No	LRT	50 Gy	Complete response

*LRT* local radiotherapy; *VP-16* etoposide; *CVAD* cyclophosphamide, vincristine, doxorubicin, dexamethasone, cytarabine, mesna, methotrexate; *CHOEP* cyclophosphamide, hydroxydaunorubicin, vincristine, etoposide, prednisone

In a palliative setting, such as in our case, the benefit of a higher radiation dose should be weighed against the comparatively long treatment time of up to over 3 weeks [12–14, 30], as longer treatment time and trips to the hospital can cause strain for patients. Moreover, in our case, systemic disease occurred before skin lesions, which developed after systemic therapy had already been initiated. The patient had only been referred to us in the described advanced stage of the disease and with multiple lesions, contributing to our choice of a hypofractionated treatment regimen.

This choice was corroborated by evidence regarding the effect of low-dose radiotherapy in other hematological malignancies such as indolent B‑cell lymphoma or chloroma and leukemia cutis, which showed a high sensitivity to even low doses. The randomized FORT trial compared the effect of 4 Gy in two fractions to 24 Gy in 12 fractions in patients with early- and advanced-stage follicular and marginal zone lymphoma [44]. Here, local control was significantly better in all subgroups when 24 Gy was applied, but overall survival and time to progression did not differ between the standard and low-dose treatment group. As toxicity such as mucositis, pain in the irradiated area, and fatigue were significantly higher in the 24-Gy group, low-dose radiotherapy could offer a short and well-tolerated treatment in palliative cases. Furthermore, if needed, re-irradiation would be feasible after 4 Gy in most cases. In case of chloroma lesions, Oertel et al. found a complete response to doses < 10 Gy and > 10 Gy/< 20 Gy in 75 and 83%, respectively [45]. Several publications report a favorable response of leukemia cutis to comparable low doses of radiotherapy, delivered either as whole-skin electron beam or focal radiotherapy [45–47]. In a consensus statement, Bakst et al. recommend doses of 24 Gy for chloroma and leukemia cutis, with cumulative doses of as low as 6 Gy in clinical settings which call for a short total treatment time [48]. In mycosis fungoides, good local control was attained by ultrahypofractionated whole-skin electron beam irradiation with 8 Gy in two fractions, resulting in a shorter treatment time and less toxicity [49, 50]. In patients with diffuse/multiple skin manifestations, total skin electron beam therapy may be required to cover the involved sites [51]. The reported publications are in line with our choice of dose and fractionation, as in our case, too, skin lesions were symptoms of an underlying systemic and generalized hematological malignancy.

More generally, a recent review on application of radiotherapy in lymphoma also supports low-dose and (ultra)hypofractionated treatment regimens in frail and palliative patients [52]. Furthermore, apart from a marked sensitivity of hematological malignancies to radiotherapy, implementing (low-dose) radiotherapy in treatment regimens involving immunotherapy and/or CAR‑T cell therapies might improve treatment by causing a priming effect in the antitumor immune system response. Radiation has been shown to result in immunogenic cell death, thereby facilitating tumor antigen release and boosting the antitumor immune response [53–55]. However, more research is needed to determine the sequence, dose, and timing needed to attain optimal results regarding the combination of systemic therapy and radiation in hematologic malignancies with skin manifestation.

In the case of the patient reported here, a short treatment concept was chosen deliberately in line with the presented data on low-dose and hypofractionated therapies in a palliative setting as the patient was treated as an outpatient with a long traveling distance to the hospital. Also, he presented with multiple lesions and systemic involvement, for which he already received hydroxyurea. Irradiated lesions responded swiftly to a comparatively low total dose of 8 Gy without reported radiation toxicity, corroborating the chosen short hypofractionated radiotherapy concept in the presented setting.

## Conclusion

Herein, we report a good and satisfactory clinical response of BPDCN skin lesions to a short hypofractionated radiotherapy with 2 × 4.0 Gy without observed toxicity. In a palliative setting, this compares favorably with regard to treatment time and attained results to published radiotherapy regimens reporting much higher doses of 27.0–51.0 Gy and a total treatment time of up to 3 weeks. Thus, palliative low-dose hypofractionated radiotherapy can serve as a viable treatment option in patients ineligible for stem cell transplantation in advanced stages of the disease, alleviating associated symptoms while minimizing the strain of treatment.
